# Clinical outcomes and unmet therapeutic needs in new-onset refractory status epilepticus: evidence from a retrospective cohort

**DOI:** 10.1186/s42466-026-00526-z

**Published:** 2026-08-28

**Authors:** Clara Jünemann, Meike Menche, Marc Philipp Bergmann, Sascha Strehlau, Panagiota-Eleni Tsalouchidou, Joane Winkel, Ole J. Simon, Lena Habermehl, Katja Menzler, Patrick Schramm, Lars Timmermann, Susanne Knake, Leona Möller

**Affiliations:** 1 Department of Neurology, Philipps University Marburg, Epilepsy Center Hessen, Philipps- University, Baldingerstr, Marburg, D-35043 Germany; 2 Second Department of Neurology, School of Medicine, ″Attikon″ University Hospital National and Kapodistrian University of Athens, Athens, Greece; 3 Department of Neurology, University Hospital Carl Gustav Carus Dresden, Dresden, Germany; 4 Center for Mind, Brain and Behavior (CMBB), Universities of Marburg, Gießen, Darmstadt, Germany; 5 LOEWE Research Cluster for Advanced Medical Physics in Imaging and Therapy, (ADMIT), Mittel Hessen University of Applied Sciences, Giessen, TH Germany

**Keywords:** Epilepsy, Status epilepticus, New onset, Refractory, NORSE

## Abstract

**Background:**

New-onset refractory status epilepticus (NORSE) is a rare, life-threatening condition occurring in individuals without prior epilepsy or acute structural, toxic, or metabolic causes. Its etiology often remains cryptogenic, and inconsistent terminology hampers early recognition and treatment. This study aims to better characterize NORSE to support faster clinical diagnosis and management.

**Methods:**

We retrospectively identified all adult patients with status epilepticus (SE) admitted to the Department of Neurology, University Hospital Marburg, Germany, between 2011 and 2023. Demographic, etiologic, treatment, and outcome data from patients fulfilling consensus criteria for NORSE were compared with those of non-NORSE patients. We additionally performed a sensitivity analysis applying strict temporal diagnostic criteria (≤ 72 h) to assess the impact of consensus-based classification on cohort definition.

**Results:**

Of 779 patients with status epilepticus, 120 fulfilled criteria for NORSE, delineating a distinct clinical subgroup characterized by advanced age and a female predominance (67.5%, *p* = 0.028). Phenotypically, NORSE was strongly associated with generalized seizure semiology (70.7%, *p* < 0.001) and a higher burden of nonconvulsive status epilepticus (45.8%, *p* < 0.001), while the underlying etiology frequently remained cryptogenic (56.7%). This constellation translated into an adverse clinical trajectory, reflected by reduced rates of discharge home, increased in-hospital mortality (19.5%, *p* < 0.001), and greater reliance on rehabilitative care. Notably, these differences were not paralleled by alterations in metabolic indices such as HbA1c or cholesterol, underscoring the limited discriminatory value of these parameters in NORSE. Application of strict temporal criteria resulted in a smaller, more conservative NORSE cohort, while key clinical features and outcomes remained largely unchanged.

**Conclusions:**

NORSE defines a distinct and severe clinical syndrome within status epilepticus, characterized by older age, female predominance and adverse outcomes. Beyond the clinical severity of NORSE, our findings show that cohort composition is highly sensitive to the temporal operationalization of consensus criteria. Standardized application of diagnostic timing may therefore be essential for comparability across NORSE studies.

## Background

 Status epilepticus (SE) is one of the most common neurological emergencies and is associated with significant mortality and morbidity [15, 16].

New-onset refractory status epilepticus (NORSE) is a rare, life-threatening condition defined by refractory SE in patients without a prior history of epilepsy and without evidence of any acute or active structural, metabolic, or toxic cause and does not represent a distinct diagnosis but instead refers to a clinical presentation encompassing a heterogeneous range of underlying etiologies of status epilepticus [13]. Despite intensive diagnostic evaluations, over 50% of cases remain cryptogenic [18], but emerging evidence suggests a potential role for immune dysregulation. With regard to cryptogenic NORSE (c-NORSE) in particular, recent studies suggest that this symptom complex arises from dysfunction of the innate immune system. Hanin & Hirsch [9], Cytokine profiling in c-NORSE patients has revealed dysregulation of chemotaxis and impaired neutrophil recruitment and migration. Nonetheless, responses to immunomodulatory or immunosuppressive therapy have shown considerable heterogeneity. Guillemaud et al., [7] If NORSE is suspected, early immunomodulatory therapy within 72 h should be considered. Hanin et al., [2, 11, 24] However, inconsistent terminology and limited awareness of the clinical syndrome continue to delay timely diagnosis and treatment.

Data on long-term outcomes in NORSE are scarce and largely confined to the in-hospital phase, with a substantial proportion of patients dying during hospitalization. Data published to date vary considerably but consistently indicate a high mortality rate (10–42%). Comparisons between NORSE and RSE further show a higher 6-month mortality rate in RSE than in NORSE overall (21.1% vs. 13.8%), whereas within the NORSE group, mortality was considerably higher in cryptogenic cases than in those with a known etiology (20% vs. 7.1%). Espino et al., [3], Hawksworth [12],, Stretti [21], NORSE differs from other forms of SE with respect to etiology, clinical course, and outcomes. This study aims to characterize NORSE within a large retrospective adult SE cohort and to assess how strict application of the 2018 consensus criteria, including the temporal dimension of diagnostic workup, affects cohort definition and the interpretation of clinical characteristics and outcomes.

## Methods

This retrospective cohort study examined the etiology, treatment patterns, and outcomes of patients with NORSE. All patients admitted to the Department of Neurology, University Hospital Marburg, Germany, between 2011 and 2023 with a primary diagnosis of SE were retrospectively reviewed based on the International League Against Epilepsy (ILAE) definition (Trinka et al., 2015). Within this cohort, patients with NORSE were identified as those with a first episode of refractory SE, no prior diagnosis of active epilepsy, and no identifiable acute structural, toxic, or metabolic cause [13]. Although NORSE was formally defined by international consensus during the study period, case classification in this study was applied retrospectively and uniformly across the entire cohort using the current consensus criteria. Original clinical diagnoses documented in the medical records were not used to determine NORSE status. Instead, all eligible cases were re-evaluated during data extraction to ensure consistent application of the definition, thereby minimizing temporal misclassification.

Exclusion criteria comprised age < 18 years, status myoclonus following hypoxic brain injury, acute hypoxic–ischemic encephalopathy, and incomplete medical records precluding reliable classification.

Clinical data were extracted retrospectively from electronic medical records using a predefined data abstraction form. Patients were identified through a structured search of the hospital information system using ICD-10 codes for status epilepticus (G41). Collected variables included demographic characteristics, NORSE classification, etiologic categories (structural, infectious, immune-mediated, metabolic, or unknown), clinical presentation (convulsive vs. nonconvulsive SE), selected laboratory and diagnostic parameters (CSF pleocytosis, C-reactive protein and lipid profile), treatment characteristics (use of intravenous anesthetics), and outcomes (modified Rankin Scale at discharge, discharge destination, and survival status at one year).

**Fig. 1:**
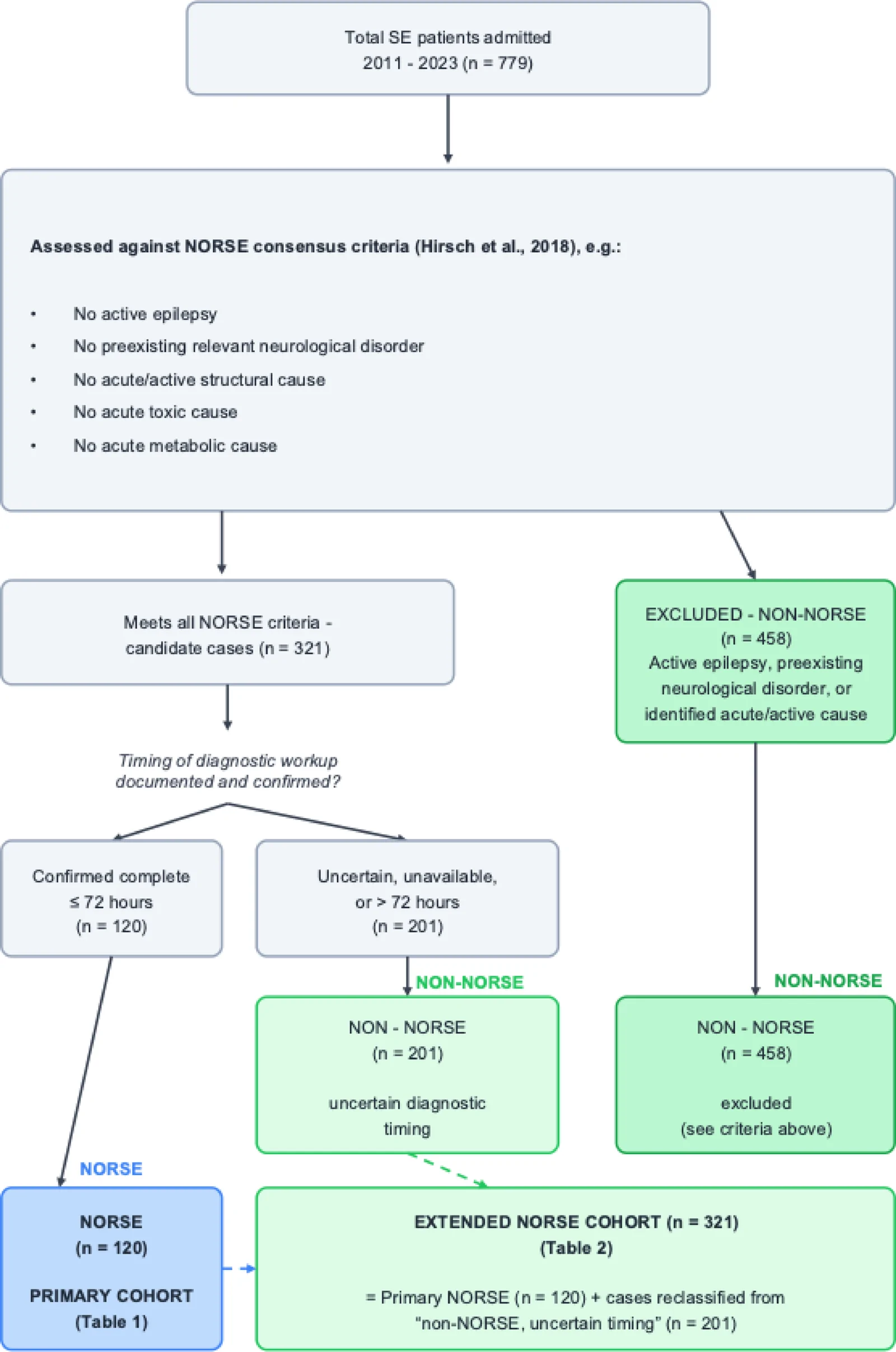
Patient Selection Flowchart

Prior to classification as NORSE, patients underwent a standardized initial diagnostic evaluation in accordance with institutional protocols and international recommendations, including neuroimaging (computed tomography and/or magnetic resonance imaging), routine blood testing, cerebrospinal fluid analysis when clinically feasible, and electroencephalography to confirm epileptic activity and distinguish convulsive from nonconvulsive SE. The scope of extended immunological testing evolved over the study period, resulting in variability in the depth of etiologic work-up, particularly in earlier years of the cohort. Follow-up data at 1 year were obtained from available hospital records, including subsequent inpatient admissions and outpatient visits at our tertiary epilepsy center, which serves as a primary referral center for the surrounding region. Patients without documented follow-up information were classified as unknown and excluded from follow-up analyses. In addition, we analyzed a larger cohort by including all patients with new-onset refractory SE, regardless of the time of diagnosis, to examine the impact of the current consensus definition, and in particular the diagnostic criteria on the clinical presentation of the cohort (Fig. 1).

Descriptive statistics are presented as absolute figures and percentages, as mean ± standard deviation for metric, normally distributed data, or as median for ordinal or non-normally distributed data. Percentages are based on available data for each variable. Cases with missing or unknown values were excluded from variable-specific analyses and percentage calculations unless otherwise specified.

Group comparisons were performed using parametric or nonparametric tests based on data distribution (SPSS version 29.0). Given the retrospective, exploratory design, no formal a priori sample size calculation was performed; the sample size was determined by the number of eligible patients treated during the study period (January 2011 - December 2023). To account for multiple testing, the Bonferroni–Holm correction was applied.

Assumptions for statistical testing were assessed prior to analysis. Normality of continuous variables was evaluated using distributional assessment and appropriate normality testing. Homogeneity of variances was assessed before applying independent t-tests; when this assumption was violated, Welch’s t-test was used. Variables not meeting assumptions of normality were analyzed using nonparametric methods. Chi-square tests were used for categorical variables when assumptions regarding expected cell frequencies were met, otherwise, Fisher’s exact test was applied.

Chi-square tests were used to compare categorical variables, independent t-tests were used for continuous variables showing normal distribution. For variables that did not meet assumptions of normality, such as length of stay (LOS), the Mann-Whitney U test was applied.

To control for potential confounding by age, separate binary logistic regression models were performed for selected variables with NORSE as the dependent variable and age included as a covariate. Results are reported as age-adjusted odds ratios with corresponding 95% confidence intervals.

Given the exploratory nature of the study and the limited number of predefined variables, no additional variables were included in the model. Multicollinearity among predictors was assessed using variance inflation factors (VIF) and tolerance statistics, with no evidence of problematic collinearity. Statistical significance was set at *p* < 0.05 (two-tailed).

The primary endpoint was survival status at one year following the index hospitalization. Secondary endpoints included functional outcome at discharge, assessed using the modified Rankin Scale, discharge destination, and selected treatment-related variables, including the use of intravenous anesthetics.

The study was approved by the Institutional Review Board of Philipps University Marburg and reported in accordance with the STROBE guidelines.

## Results

A total of 779 patients with SE were identified, of whom 120 (15.4%) met the criteria for NORSE. Among NORSE patients, the mean age was 73.5 ± 13.5 years (*p* < 0.001, OR = 1.03, vs. Non-NORSE 63.3 ± 19.1 years), with 67.5% female (*p* = 0.028, OR = 0.62, vs. Non-NORSE 48.7% female). In most NORSE cases, the etiology remained cryptogenic (56.7%), while immune-mediated (15.0%), infectious (8.3%) and possible structural (18.3%) causes were less frequent (Table 1; Fig. 3a).

**Table 1:** Diagnostic Characteristics of NORSE Patients

Characteristic	NORSE (*n* = 120)
Etiology, n (%)	
cryptogenic	68 (56.7)
structural DD cryptogenic CJD Remote brain injury	22 (18.3) 1 (4.5) 21 (95.5)
paraneoplastic	2 (1.7)
infectious HSV Klebsiella pneumoniae CMV SARS-CoV-2 no pathogen detected	10 (8.3) 1 (10.0) 1 (10.0) 1 (10.0) 2 (20.0) 5 (50.0)
immune-mediated Neurexin 3a GAD Titin no antibody detected	18 (15) 1 (5.6) 1 (5.6) 1 (5.6) 15 (83.3)
Cerebrospinal fluid diagnostics	
no abnormalities	33 (27.5)
monoclonal bands	10 (8.3)
oligoclonal bands	2 (1.7)
Identical bands	2 (1.7)
barrier dysfunction	18 (15.0)
unknown/incomplete report	55 (45.8)
Immunosuppressive Therapy	
high-dose methylprednisolone	5 (55.5)
prednisolone	2 (22.2)
hydrocortisone	1 (11.1)
Azathioprine (preexisting)	1 (11.1)
Time until LP	
< 24 h	28 (23.3)
24–48 h	14 (11.7)
48–72 h	76 (63.3)
Not performed	2 (1.7)
Time until Blood Sample	
< 24 h	120 (100.0)
24–48 h	0 (0.0)
48–72 h	0 (0.0)

CJD, Creutzfeldt-Jakob disease; CMV, Cytomegalovirus; DD, differential diagnosis; GAD, Glutamic acid decarboxylase; HSV, Herpes simplex virus; LP, Lumbar puncture

### Outcome

**Fig. 2:**
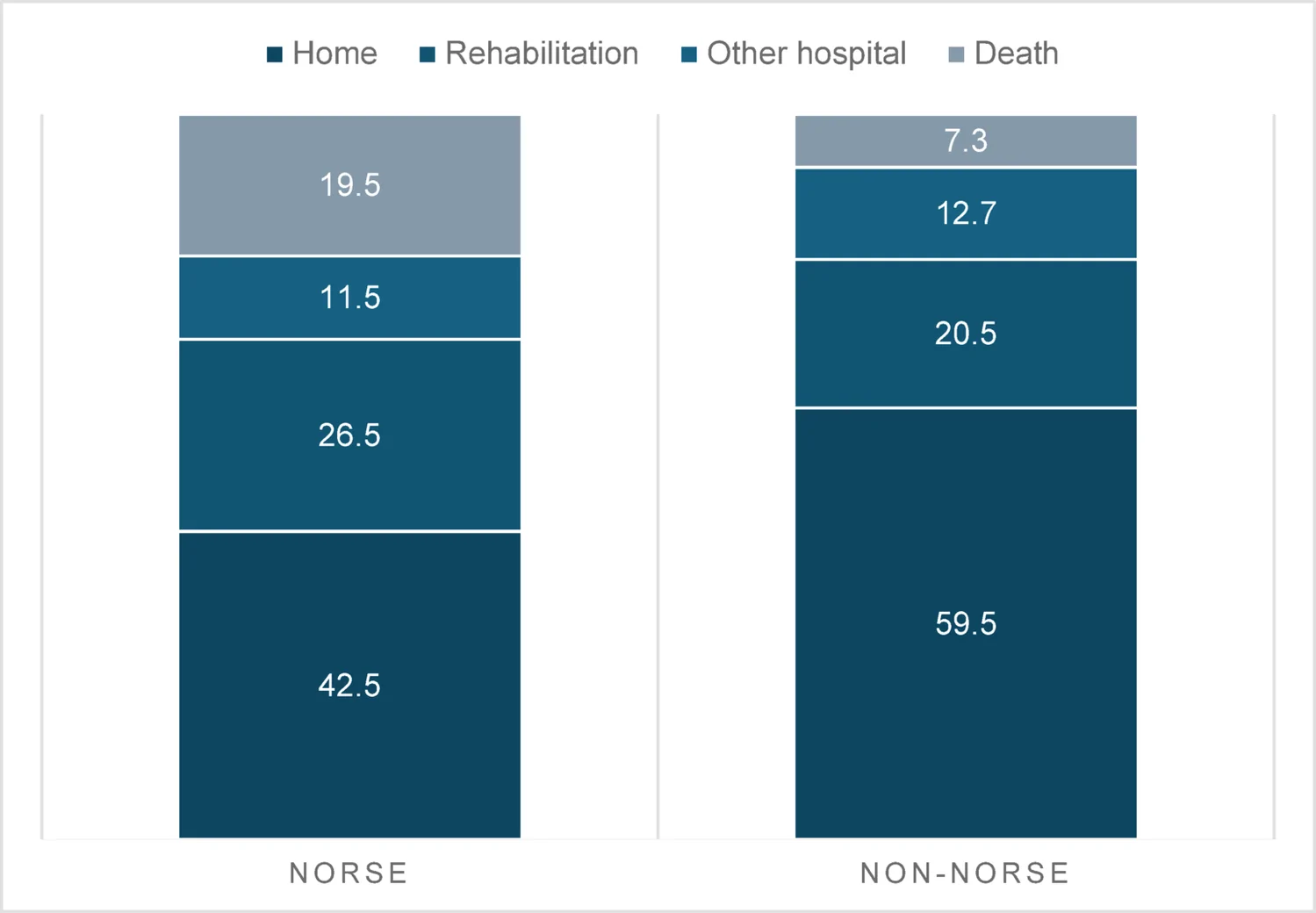
Discharge Destination of NORSE Patients in %.NORSE, new - onset refractory Status Epilepticus

The one-year survival rate was significantly lower in the NORSE cohort compared with non-NORSE patients (57.7.% vs. 81.6%, *p* = 0.002). Nonconvulsive SE occurred more frequently in NORSE patients than in non-NORSE patients (*p* < 0.001), furthermore the semiology of SE was associated with NORSE, generalized SE occurred more often with NORSE than focal SE (*p* < 0.001). Outcome at discharge, assessed by the modified Rankin Scale (mRS), was worse in the NORSE group (mRS > 2 in 82.1% vs. 71.0% in non-NORSE). NORSE patients were also less likely to be discharged home (*p* = 0.008; Table 2; Fig. 2).

**Table 2:** Characteristics and Outcome of NORSE and Non-NORSE Patients according to the current definition

Patient Characteristics	NORSE (*n* = 120)	Non-NORSE (*n* = 659)		*p*-value		Age adjusted *p*-value	OR, 95% - CI
Demographics							
Age, mean ± SD (range)	73.5 ± 13.5 (23–96)	63.3 ± 19.1 (18–95)		< 0.001			1.03, 1.02–1.05
Sex, n (%)				< 0.001		**0.028**	0.62, 0.41–0.95
Male	39 (32.5)	337 (51.1)					
Female	81 (67.5)	321 (48.7)					
Semiology				< 0.001		**< 0.001**	0.25, 0.16–0.40
Focal	29 (29.3)	356 (58.0)					
Generalized	70 (70.7)	258 (42.0)					
Nonconvulsive SE (NCSE)	55 (45.8)	110 (16.7)		< 0.001		**< 0.001**	4.16, 2.61–6.62
Convulsive	65 (54.2)	549 (83.3)					
Diagnostics and Therapy, n (%)							
Pleocytosis (≥ 5/µl) No Pleocytosis	10 (16.1) 52 (83.9)	26 (13.5) 167 (86.6)		0.601		0.423	1.40, 0.61–3.23
CRP ≥ 5 mg/L < 5 mg/dl	64 (55.2) 52 (44.8)	300 (45.8) 355 (54.2)		0.062		0.288	1.25, 0.83–1.89
Use of intravenous anesthetics No use	20 (42.6) 27 (57.4)	106 (43.4) 138 (56.6)		0.968		0.684	1.14, 0.66–1.97
Outcome, n (%)							
mRS at discharge				0.067		**0.515**	1.27, 0.62–2.57
≤ 2	12 (17.9)	91 (29.0)					
> 2	55 (82.1)	223 (71.0)					
Length of inpatient stay, mean ± SD (median), days	12.4 ± 9.4 (10.0)	12.3 ± 12.5 (8.0)		0.079		0.825	0.998, 0.98–1.02
Discharge destination, n (%)				< 0.001		**0.008**	1.73, 1.15–2.60
Home	48 (42.5)	374 (59.5)					
Rehabilitation	30 (26.5)	129 (20.5)					
Other hospital	13 (11.5)	80 (12.7)					
Death	22 (19.5)	46 (7.3)					
Status at 1 year, n (%)			< 0.001		0.002		2.28, 1.34–3.88
Alive	45 (57.7)	369 (81.6)					
Dead	33 (42.3)	83 (18.4)					
Cholesterol							
Total > 200 mg/dl	32 (26.7)	114 (17.3)	0.049		0.069		0.61, 0.36–1.04
HDL ≤ 40 mg/dl	16 (13.3)	86 (13.1)	0.942		0.966		1.01, 0.53–1.92
LDL > 116 mg/dl	34 (28.3)	145 (22.0)	0.107		0.227		0.69, 0.38–1.26
Statin intake	36 (30.0)	168 (25.5)	0.424		0.695		0.91, 0.58–1.42
Diabetes known	30 (25.0)	141 (21.4)	0.848		0.746		0.92, 0.58–1.46
Hba1c			0.550		0.676		1.20, 0.82–1.75
≤ 5.7	20 (54.1)	100 (59.5)					
5.7–6.4	11 (29.7)	36 (21.4)					
> 6.4	6 (16.2)	32 (19.0)					

CSF, cerebrospinal fluid; CRP, C-reactive protein; HDL, high-density lipoprotein; LDL, low-density lipoprotein; mRS, Modified Rankin Scale; NCSE, nonconvulsive status epilepticus; SE, status epilepticus

P-values were calculated using χ² or Fisher’s exact test for categorical variables and independent-samples t-test or Mann–Whitney U test for continuous variables, as appropriate. Adjusted analyses were performed using binary logistic regression including age as a covariate. Statistically significant values (p < 0.05) are highlighted in bold

### Diagnostics and treatment

Analysis of diagnostic parameters revealed no significant association between NORSE occurrence and inflammatory markers in cerebrospinal fluid (cell count, autoantibodies when available) or serum (CRP). Similarly, the analysis of metabolic parameters, such as cholesterol levels, statin use, or metabolic disorders, showed no correlation with NORSE. Due to the retrospective study design, not all cerebrospinal fluid (CSF) biomarkers could be comprehensively assessed, particularly in the earliest cases. Among the available results, most were unremarkable, blood-brain barrier dysfunction as well as oligoclonal and monoclonal bands were identified in a subset of patients. Blood sampling was performed within 24 h of SE onset in 100% of NORSE cases, whereas CSF sampling was most often performed within 72 h (63.3%), and within the first 24 h in approximately 23% of cases. Data on immunosuppressive therapy were available for only a small proportion of patients, among these, high-dose methylprednisolone (1000 mg/day) was the predominant regimen, accounting for 55.5% of immunosuppressive treatments.

In the NORSE group, intravenous anesthetics were not used more frequently than in non-NORSE patients. The length of hospital stay was also comparable.

### Results of the NORSE cohort defined without strict temporal criteria

Among 779 patients with SE, 321 (41.2%) were classified as NORSE when including cases with uncertain or unavailable timing of diagnostic assessments (Table 3). The Non-NORSE cohort had a mean age of 64.9 ± 18.9 years, with 47.4% females. NORSE patients were similar (66.0 ± 18.7 years; 52.0% female).

**Table 3:** NORSE cohort defined without strict temporal criteria

Patient Characteristics	NORSE (*n* = 321)	Non-NORSE (*n* = 458)
Demographics		
Age, mean ± SD (range)	66.0 ± 18.7 (18–94)	64.0 ± 18.9 (19–96)
Sex, n (%)		
Male	120 (37.4)	240 (52.4)
Female	201 (62.6)	217 (47.4)
unknown	0 (0.0)	1 (0.2)
Etiology, n (%)		
Structural	66 (20.6)	80 (17.5)
Infectious	47 (14.6)	171 (37.4)
Immune-mediated	3 (0.9)	5 (1.1)
Metabolic	3 (0.9)	16 (3.5)
Unknown	202 (63.0)	186 (40.6)
Diagnostics and Therapy, n/N (%)		
Nonconvulsive SE (NCSE)	90/321 (28.0)*	75/458 (16.4)
Pleocytosis (CSF)	23/157 (14.6)	13/98 (13.3)
CRP > 5 mg/L	164/318 (51.6)	199/455 (43.7)
Sodium ≤ 125 mmol/L	10/321 (3.1)	11/458 (2.4)
Use of intravenous anesthetics	49/70 (67.1)*	77/220 (35.0)
Outcome, n (%)		
mRS ≤ 2 at discharge	61 (29.3)	42 (24.4)
mRS > 2 at discharge	147 (70.7)	130 (75.6)
Length of inpatient stay, mean ± SD (median), days	12.3 ± 10.4 (9)	13.2 ± 12.1 (9)
Discharge destination, n (%)		
Home	144 (46.9)*	281 (47.6)
Rehabilitation	78 (25.4)	81 (18.5)
Other hospital	39 (12.7)	54 (12.4)
Death	46 (15.9)	21 (4.8)
Status at 1 year, n (%)		
Alive	146 (67.6)	268 (85.4)
Dead	70 (32.4)	46 (14.6)

* *p* < 0.001

In NORSE, etiology remained unknown in most cases (62.9%), followed by structural (20.6%) and infectious causes (14.6%).

One-year survival was significantly lower in NORSE compared with non-NORSE patients (67.6% vs. 85.4%, *p* < 0.001). Nonconvulsive SE occurred more frequently in NORSE (28.0% vs. 16.4%, *p* < 0.001), although convulsive SE remained the predominant presentation in both groups (70.7% vs. 75.6%). Functional outcome at discharge was worse in NORSE patients, with a higher proportion achieving mRS > 2 (64.5% vs. 54.8%). Consistently, NORSE patients were less likely to be discharged home (*p* < 0.001).

Diagnostic analyses showed no significant associations between NORSE and inflammatory markers in cerebrospinal fluid or serum (including CRP and autoantibodies when available).

Treatment intensity differed substantially between groups: intravenous anesthetics were used significantly more often in NORSE patients than in non-NORSE patients (67.1% vs. 35.0%, *p* < 0.001).

## Discussion

In this retrospective SE cohort, strict application of the 2018 consensus criteria identified NORSE as a clinically severe subgroup with poor outcome. At the same time, our comparison of cohorts with and without strict temporal diagnostic constraints indicates that the operationalization of NORSE criteria itself substantially influences cohort composition. This methodological aspect may be critical for interpreting and comparing published NORSE studies.

Among 779 patients with SE, 15.4% fulfilled criteria for NORSE, most commonly in the absence of an identifiable etiology. NORSE was associated with female sex, a predominance of generalized and nonconvulsive seizure patterns, and poorer clinical outcomes. The high burden of nonconvulsive presentations in NORSE underscores the diagnostic and prognostic challenges of NCSE in critically ill, often unconscious patients, for whom standardized diagnostic criteria and outcome prediction tools have recently been proposed Willems et al., [25].

Population-based estimates of NORSE incidence and prevalence remain unavailable. However, cohort studies indicate that NORSE constitutes a substantial subset of refractory status epilepticus, accounting for up to approximately 20% of cases, with a reported proportion of 16% in a prospective pediatric cohort [19, 21]. In the present study, NORSE was defined according to international consensus criteria as refractory status epilepticus without an identifiable acute or active structural, toxic, or metabolic cause [13]. The retrospective design spanning a decade, during which diagnostic capabilities, particularly in earlier years, were more limited, may have increased the proportion of cases classified as cryptogenic. In addition, the role of our institution as a tertiary epilepsy referral center with a dedicated neurological intensive care unit likely resulted in preferential admission of more severe and refractory cases, thereby influencing the observed prevalence. The demographic characteristics of the overall cohort were consistent with prior adult studies of SE [14, 15], in which younger age, acute symptomatic etiology, and impaired level of consciousness have been identified as predictors of refractory status epilepticus [22].

### Demographics and risk factors

In this cohort, NORSE affected older patients, a finding that contrasts with previous reports identifying younger age as a predictor of refractory status epilepticus (RSE) [22]. While status epilepticus overall increasingly affects older individuals, prior studies have suggested a distinct demographic profile for NORSE, with a relative predominance among younger adults. Our findings therefore highlight a notable discrepancy that may reflect the tertiary care setting with referral of more severe and comorbid cases, the adult-only cohort, and the contribution of age-associated etiologies such as structural and degenerative conditions.

In line with previous reports, female sex appeared to be associated with NORSE. Stretti et al., [21]. Since an autoimmune cause is suspected in many cases and NMDA receptor encephalitis is often found in confirmed cases, this could explain the higher proportion of women.Hawksworth et al., [12]

### Etiology

Despite comprehensive diagnostic evaluation, the etiology remained unknown in 56.7% of NORSE patients, comparable with commonly reported [6, 12]. Among identifiable causes, infectious and structural etiologies were most common. Although consensus-based recommendations for the management of NORSE have been established only recently, clinical practice remains heterogeneous, reflecting the prior reliance on case series and limited evidence [24]. As a result, NORSE may be recognized late in its clinical course, potentially delaying the initiation of appropriate therapeutic strategies in a condition characterized by rapid progression and poor prognosis.

### Inflammation and autoimmunity

The role of autoimmunity in NORSE remains incompletely understood. While antibody-negative autoimmune encephalitis has been proposed as a potential mechanism in a subset of cryptogenic cases, recent data highlight substantial diagnostic uncertainty [5].

In our cohort, conventional inflammatory markers in serum and cerebrospinal fluid did not distinguish NORSE from non-NORSE SE, however, the conclusions are limited due to the small number of cases. Pleocytosis was observed only in a minority of patients, and systemic inflammatory markers showed no relevant associations. These findings should be interpreted with caution given the retrospective design and variability in diagnostic workup. While not supporting a strong association in this setting, they do not exclude a role of inflammatory or immune-mediated mechanisms.

Taken together, our results argue against a uniform and readily identifiable autoimmune etiology but are compatible with a more heterogeneous and potentially subtle immune contribution in selected cases.

### Cholesterol and lipid metabolism

Recent studies have explored potential links between lipid metabolism, statin therapy, and seizure activity, including possible preventive or therapeutic effects [1, 8]. In addition, the ketogenic diet has been investigated as a treatment approach in NORSE and refractory SE, although current evidence remains insufficient to support its routine use [4, 17]. Statin therapy prior to status epilepticus onset has been associated with reduced mortality in prospective cohorts, potentially mediated through anti-inflammatory mechanisms, although findings remain inconsistent [20, 23].

In the present cohort, lipid parameters and statin use were not clearly associated with NORSE. Notably, NORSE patients did not exhibit the typical cardiovascular risk profile observed in status epilepticus of vascular origin, suggesting that lipid-related mechanisms may differ fundamentally between these populations. However, the timing of sample collection was not systematically accounted for, which limits the interpretability of this comparison. Findings from Hanin and colleagues suggest that status epilepticus may induce inhibition of CYP46A1, with the resulting cholesterol accumulation in the brain potentially contributing to neurological sequelae through excitotoxic mechanisms. Hanin et al., [10]Overall, our findings do not support a major role of lipid metabolism in NORSE but highlight the need for prospective studies to further explore this potential association.

### Treatment and outcomes

NORSE was associated with significantly poorer outcomes, reflected by lower rates of discharge home and reduced one-year survival. Although a proportion of patients survived, outcome differences were more pronounced in the NORSE cohort. While a recent study suggest that functional recovery may occur despite initially poor modified Rankin Scale scores, such an association was not evident in our cohort. Furthermore, this Swiss study found no significant differences in baseline characteristics between NORSE patients who survived and those who died during hospitalization, underscoring the severity and clinical unpredictability of the syndrome Stretti et al., [21].

These findings also highlight the acute severity of NORSE and its significant impact on both short- and long-term outcomes. Notably, despite recommendations for immunomodulatory or immunosuppressive therapy, such treatment was administered in only a minority of NORSE cases. This finding underscores, on one hand, the heterogeneous management of this condition in clinical practice and the limited implementation of existing recommendations. On the other hand, it should be noted that our study period dates back to 2011, whereas understanding and awareness of NORSE have grown considerably in recent years. Furthermore, documentation inherent to the retrospective design likely represents an additional limitation, potentially introducing bias.

### Methodological considerations in NORSE classification

Importantly, applying the consensus criteria proposed by Hirsch et al., [13] with explicit consideration of the temporal dimension of diagnostic workup had a substantial impact on cohort composition. In our study, the cohort defined by strict adherence to the 72-hour diagnostic window most closely reflects the current consensus-based understanding of NORSE and therefore serves as the primary analytical framework.

The comparison with a broader cohort, in which cases were included irrespective of the timing of diagnostics, highlights how strongly NORSE classification depends on the operationalization of these criteria. While this extended cohort yielded a markedly higher proportion of NORSE cases (41.2%) and differed in demographic characteristics, key clinical features such as etiological distribution and outcome remained largely unchanged.

This suggests that the temporal specification of diagnostic workup primarily influences epidemiological estimates and cohort composition, whereas core clinical characteristics of NORSE appear more robust. These findings underline the importance of clearly defining and transparently reporting the application of consensus criteria when interpreting and comparing NORSE studies.

### Limitations and future directions

This study has several limitations. Its retrospective, single-centre design limits generalizability, and cardiovascular risk factors as well as the impact of prior or concurrent therapies were not systematically assessed. Furthermore, although a standardized initial diagnostic evaluation was performed, the extent of advanced etiologic testing, particularly autoimmune and paraneoplastic investigations, varied over time, reflecting evolving diagnostic availability and clinical practice. This variability may have influenced the likelihood of identifying specific etiologies.

Nevertheless, the large cohort size and the broad catchment area of our tertiary referral center provide a robust basis for characterizing NORSE within an adult population. Future multicenter, prospective studies are required to further delineate underlying mechanisms and to refine diagnostic and therapeutic strategies aimed at improving outcomes.

## Conclusion

NORSE represents a severe clinical presentation within SE, characterized in our cohort by older age, female predominance, and a high proportion of generalized and nonconvulsive seizure patterns. Compared with non-NORSE status epilepticus, patients exhibited significantly worse outcomes, including reduced survival and lower rates of discharge home. The predominance of cryptogenic cases further underscores the heterogeneity of this condition. These findings highlight the clinical relevance of recognizing NORSE as a distinct presentation associated with an adverse prognosis and specific demographic and phenotypic features.

**Fig. 3:**
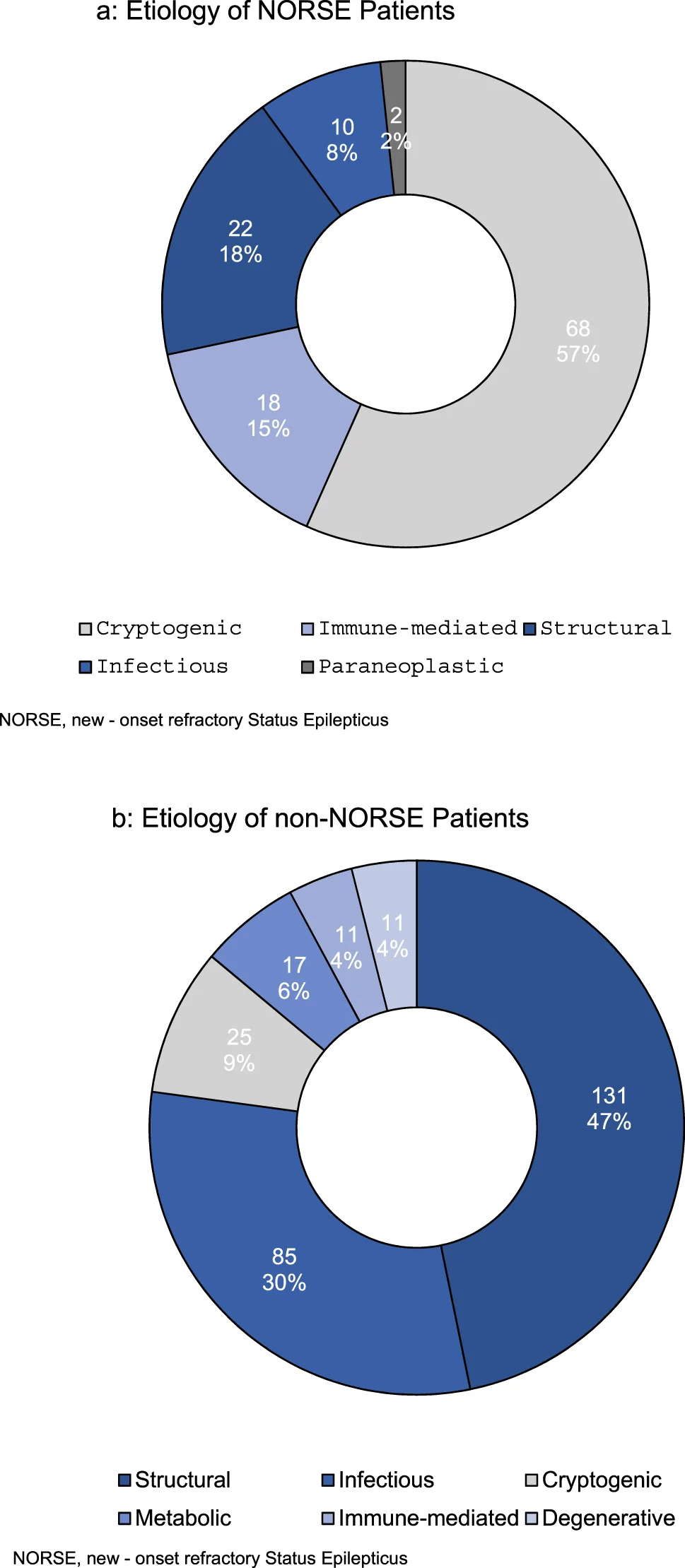
**a**: Etiology of NORSE Patients.NORSE, new - onset refractory Status Epilepticus. **b**: Etiology of non-NORSE Patients. NORSE, new - onset refractory Status Epilepticus

## Data Availability

The data supporting the findings of this study are not publicly available due to privacy and ethical restrictions involving patient data but are available from the corresponding author on reasonable request and subject to institutional and ethical approval.

## Abbreviations

CRP: C-reactive protein
CSF: Cerebrospinal fluid
DRE: Drug Resistant Epilepsy
HDL: High Density Lipoprotein
ICU: Intensive Care Unit
ILAE: International League Against Epilepsy
IRB: Institutional Review Board
LDL: Low Density Lipoprotein
LOS: Length of Stay
mRS: Modified Rankin Scale
NCSE: Nonconvulsive Status Epilepticus
NORSE: New - Onset Refractory Status Epilepticus
NRSE: Non-Refractory Status Epilepticus
RSE: Refractory Status Epilepticus
SD: Standard deviation
SE: Status Epilepticus
SRSE: Super Refractory Status Epilepticus
